# Knowledge, attitudes, and practices regarding urinary tract infections among women in the United Arab Emirates

**DOI:** 10.1371/journal.pone.0298993

**Published:** 2025-01-10

**Authors:** Shaikha Salah Alhaj, Sajad Allami, Amjad Mohamadiyeh, Ammar Agha, Abdul Kareem Abu Ali, Joudi M. Bassam Habbal, Balsam Qubais Saeed

**Affiliations:** 1 College of Medicine, University of Sharjah, Sharjah, UAE; 2 Clinical Sciences Department, College of Medicine, University of Sharjah, Sharjah, UAE; Hawassa University College of Medicine and Health Sciences, ETHIOPIA

## Abstract

**Background:**

Urinary tract infections (UTIs), which are infections of the kidneys, ureters, bladder, or urethra, are a worldwide public health concern. As compared to men, women are more prone to UTIs. There have been several studies that explore the knowledge, attitudes, and practices of women regarding UTIs in different countries, but no such study has been conducted in the UAE; therefore, we conducted this study in the UAE setting.

**Methods:**

This study was conducted using an online survey created on Microsoft Forms. The minimum sample size required for our study was 385. This study was conducted after obtaining ethical approval from the Research Ethics Committee at the University of Sharjah. A personally designed questionnaire consisting of 21 items, derived from previous research was used to record data. The data was analyzed using SPSS.

**Results:**

A total of 475 females were included in the study. Most respondents were aged 18–30 years (47.4%). Our study found that a majority of the participants (69.7%) correctly identified bacteria as the most common cause of UTIs. With regards to practices undertaken during UTIs, among the participants with a history of UTI episodes, 32.6% waited 24–48 hours before seeking medical attention at a hospital or clinic, while 10% did not visit a hospital at all. Distinct trends were found when comparing demographic factors with knowledge levels. Most notably, the age group of 18–30 years showed the highest percentage of high-knowledge individuals (49%) compared to other age groups (p < 0.05). Education level was significantly (p = 0.003) associated with UTI knowledge. Going to the hospital/clinic was reported by 41% with high knowledge but only by 20% of those with poor knowledge. Moreover, a higher proportion of individuals with high knowledge sought medical attention immediately within 24 hours (47%).

**Conclusion:**

Most of the participants possessed adequate knowledge regarding UTIs. Higher knowledge levels were associated with more proactive and appropriate health behaviors, such as seeking medical attention promptly and drinking more water.

## Introduction

Urinary tract infections (UTIs), which are infections of the kidneys, ureters, bladder, or urethra, are a worldwide public health concern [1]. According to a study, over 404.6 million people all around the globe had UTIs in 2019, and nearly 236,786 died as a result of UTIs the same year [2]. As compared to men, women are more prone to UTIs [1]. One of the reasons attributable to this fact is the presence of shorter urethra in women, which makes it easier for bacteria or other microbes to reach the bladder or urinary tract and cause infection [1, 3]. It is estimated that approximately 60% of females experience at least one UTI in their lifetime [3], which is more likely to occur in menopausal or pregnant women [3, 4].

The urinary system possesses various mechanisms to prevent foreign bacteria from entering; however, when these mechanisms fail to eliminate these bacteria UTIs can occur. *Escherichia coli* is the most associated bacteria with UTIs. Other causative pathogens include *Staphylococcus saprophyticus*, *Proteus*, *Klebsiella*, and *Pseudomonas aeruginosa* [5, 6]. Additionally, certain risk factors predispose women to UTIs, which include pregnancy, sexual activity (new sexual partner), menopause, use of certain birth controls, catheters, or a suppressed immune system [5, 6]. UTIs can be both symptomatic and asymptomatic. Symptomatic UTIs are characterized by painful and frequent urination, hematuria, suprapubic pain, and fever [4, 5]. On the other hand, asymptomatic UTIs do not have any apparent symptoms but are characterized by the presence of bacteria in urine [5, 7]. However, both symptomatic and asymptomatic infections should be treated [7] to avoid further complications such as recurrent infections, kidney damage, bladder dysfunction, or preterm labor [6, 8].

Despite being preventable, UTIs are quite prevalent among women in the UAE [4]. They can be prevented by staying well-hydrated, maintaining hygiene, urinating after sexual activity, and raising awareness among girls and women [1, 6]. There have been several studies that explore the knowledge, attitudes, and practices of women regarding UTIs in different countries [9, 10], but no such study has been conducted in the UAE; therefore, in this study, we aim to explore the knowledge, attitudes, and practices of women in the UAE regarding UTIs. The results of this study will aid us in determining the level of awareness among them, which can contribute to targeted awareness campaigns and educational initiatives related to women’s health.

## Methods

The Strengthening the Report of the Observational Studies in Epidemiology (STOBE) guidelines were used throughout conducting this study [11].

### Study design

This study was a cross-sectional survey to assess the knowledge, attitude, and practices (KAP) regarding Urinary Tract Infections (UTI) among women in the United Arab Emirates (UAE).

### Study settings and selection of participants

This study was conducted using an online survey created on Microsoft Forms. The minimum sample size required for our study was 385, collected using the Raosoft Sample Size Calculator with a confidence interval of 95%, a margin of error of 5%, and a population size of 3.2 million. The data for the population size of women in the UAE was used according to the latest population statistics [12]. However, we calculated data from 475 participants. The inclusion criteria for participants included: i) females of any age; ii) residents of the UAE. The exclusion criteria included: i) males; ii) not a resident of the UAE.

### Ethics statement

Data was collected from participants after obtaining informed consent. The online consent was taken prior to filling out the questionnaire. A brief summary of the questionnaire ahead was provided along with the purpose of filling out this form. It was clarified that participation is anonymous and voluntary. Only those who consented to participate could access the form ahead. In the case of minors, it was mandatory that the consent form was filled out in the presence of parents or guardians. Participants were ensured that their information would be kept confidential. This study was conducted after obtaining ethical approval from the Research Ethics Committee at the University of Sharjah (Reference Number: REC-23-09-19-01-F).

### Data collection and statistical analysis

A personally designed questionnaire consisting of 21 items, derived from previous research and expert opinion [9, 10], was used to record data. A pilot study was also conducted to further validate the questionnaire, ensuring that the questions were understandable, relevant, and effectively captured the intended data. It was divided into four sections: i) demographics details; ii) questions to determine knowledge of the respondents; iii) questions to determine the attitude of the respondents; iv) questions to determine practices of the respondents. Both English and Arabic versions of the questionnaire were used to collect data. Data collection was started on 24^th^ October 2023 and we completed collecting data on 1^st^ December 2023.

The data was collected and coded. It was then analyzed using IBM Statistical Package for Social Sciences (SPSS) for Windows, Version 21 (IBM Corp, Armonk, NY, USA). A descriptive analysis was run. A knowledge score was calculated, where a 0–1 score was categorized as poor knowledge, a 2–3 score was categorized as moderate knowledge, and a 4–6 score was categorized as high knowledge. To study the association of factors with knowledge, a chi-square test was run, and a p < 0.05 was used as the level of significance.

## Results

### 1. Characteristics of the study population

A total of 475 females were included in the study. Most respondents were aged 18–30 years (47.4%). The participants were equally distributed between married (48.8%) and single (48.2%), with a small minority divided between divorced and widowed. A significant majority of the participants held a bachelor’s degree (66.9%), followed by those with a high school diploma (18.1%). Most participants lived in Sharjah and Dubai (73.1%), while the rest were distributed among the remaining emirates. 35 participants (7.3%) had either diabetes or hypertension. A summary of the demographic characteristics of the participants is shown in Table 1.

**Table 1:** Demographic characteristics of the participants.

Variable	N	%
**Age**		
<18	16	3.4
18–30	225	47.4
31–40	113	23.8
41 and above	121	25.5
**Marital Status**		
Divorced	6	1.3
Married	232	48.8
Single	229	48.2
Widow	8	1.7
**Educational Level**		
Bachelor’s degree	318	66.9
Post Graduate Studies	66	13.9
High school	86	18.1
Primary/middle school	5	1.1
**Emirate**		
Abu Dhabi	88	18.5
Ajman	31	6.5
Dubai	122	25.7
Fujairah	4	0.8
Ras Al Khaimah	3	0.6
Sharjah	225	47.4
Um al Quwain	2	0.4

### 2. Knowledge of the participants regarding urinary tract infections

Participants who responded ’No’ to the question “Have you heard of the term urinary tract infection (UTI)?” were categorized as possessing a minimal level of knowledge (18.7%), and the questionnaire was subsequently concluded for them at that point. The most prevalent understanding of UTIs among these respondents is inflammation of the kidney, bladder, or urethra (53.5% chose “Can be in all the above”), followed by ’Inflammation of the urethra’ only (23.2%). A majority (69.7%) correctly identify bacteria as the most common cause of UTIs. Additionally, most participants were able to accurately identify the symptoms, habits that increase the risk, and preventive measures of UTIs. Table 2 provides a detailed breakdown of the participants’ knowledge regarding urinary tract infections, including the number and percentage of responses for each question.

**Table 2:** Knowledge of the participants regarding urinary tract infections.

Variable	N	%
**Have they heard of the term UTI?**		
No	89	18.7
Yes	386	81.3
**Definition of UTI**		
Inflammation of the bladder	17	4.4
Inflammation of the kidney	2	0.5
Inflammation of the urethra	110	28.5
None of the above	3	0.8
All of the above*	254	65.8
**Most common cause of UTI**		
Bacteria*	269	69.7
Bad hygiene	37	9.6
Fungi	58	15.0
Viruses	22	5.7
**Which symptom/s do you think occur with UTI?**		
Pain during urination*	351	19.5
Change in the urine color/appearance*	302	16.8
Abdominal pain*	205	11.4
Back pain*	170	9.5
Increase in the frequency of urination*	251	14.0
Leg pain	31	1.7
Fever*	199	11.1
Constipation	22	1.2
A sudden desire to go to bathroom to urinate*	266	14.8
**Which habit/s do you think increases the chances of having UTI?**		
Cleaning the genitalia from back to front*	238	27.5
Drinking a small amount of water*	334	38.6
Delaying urination*	267	30.8
Urinating after sexual intercourse	10	1.2
Cleaning the genitalia from front to back	9	1.0
Drinking large amounts of water	8	0.9
**Which factor/s do you think prevent UTI?**		
Cleaning the genitalia from back to front	20	2.3
Drinking a small amount of water	3	0.3
Delaying urination	5	0.6
Urinating after sexual intercourse*	207	23.8
Cleaning the genitalia from front to back*	278	31.9
Drinking large amounts of water*	358	41.1

* represents the correct answers

### 3. Attitude of the participants regarding urinary tract infections

Most respondents (69.6%) believe that going to the hospital/clinic is the appropriate way to deal with a UTI. Drinking more water is also a common response, with 19.2% of respondents suggesting this method. 46.1% of respondents perceive UTI as a serious condition/disease. Some of the most commonly believed complications of UTIs included “damage to the kidneys” and “decrease in quality of life” (39.9% and 23.6%, respectively). The participants’ attitudes toward urinary tract infections, including the number and percentage of responses for each item, are detailed in Table 3.

**Table 3:** Attitude of the participants regarding urinary tract infections.

Variable	N	%
**In your opinion, what is the appropriate way of dealing with UTI?**		
Drink more water	74	19.2
Go to the Hospital/Clinic	269	69.6
I don’t know	9	2.3
Rest at home	1	0.3
Shower more frequently	6	1.6
Take analgesics (painkillers)	1	0.3
Take antibiotics directly	26	6.7
**Do you think UTIs are common?**		
Yes	363	94
No	23	6
**Which of the following statements do you think is true regarding UTI?**		
I don’t know	34	8.9
UTIs affect both genders equally	52	13.5
UTIs affect females more	295	76.3
UTIs affect males more	5	1.3
**Which complications do you expect from having a UTI?**		
It can damage the kidneys	289	39.9
It can decrease the quality of life	171	23.6
It can lead to recurrent UTIs	142	19.6
It can affect pregnancies	85	11.8
It can lead to death	29	4
It can cause weight loss	8	1.1
**Do you feel UTI is a serious condition/disease?**		
I don’t know	70	18.1
Yes	178	46.1
No	138	35.8

### 4. Practices of the participants regarding urinary tract infections

With regards to practices undertaken during UTIs, among the participants with a history of UTI episodes, 56 (32.6%) waited 24–48 hours before seeking medical attention at a hospital or clinic, while 17 (10%) did not visit a hospital at all. The most common initial actions taken by these participants included visiting a hospital or clinic and increasing water intake (48% and 38.5%, respectively). The practices of the participants regarding urinary tract infections, along with the number and percentage of responses for each practice, are summarized in Table 4.

**Table 4:** Practices of the participants regarding urinary tract infections.

Variable	N	%
**Have you ever had a UTI before?**		
Yes	172	44.6
No	214	55.4
**What did you do when you felt symptoms of UTI?**		
Drank more water	114	38.5
Go to the hospital/clinic	142	48.0
Had rest at home	6	2.0
Took antibiotics directly	8	2.7
Took more showers	5	1.7
Took analgesics (painkillers)	19	6.4
I don’t remember	2	0.7
**If yes, how long did you wait before visiting the hospital/clinic?**		
< 24 hours	49	28.4
24–48 hours	56	32.6
2–5 days	32	18.6
More than 5 days	18	10.4
I did not go to the hospital	17	10
**Which symptoms do you have?**		
Pain during urination	129	23.5
Change in the urine color and appearance	83	15.1
Back pain	55	10.0
Increase in the frequency of urination	94	17.2
A sudden desire to go to bathroom to urinate	87	15.9
Fever	33	6.0
Abdominal pain	67	12.2
**How much water do you drink per day?**		
< 0.5 Litres	34	8.8
0.5 Litres to 1 Litre	159	41.2
1 Litre to 2 Litres	142	36.8
More than 2 Litres	51	13.2

### 5. Outcomes of consultation

Regarding participants’ expectation of consultation outcomes, participants were required to rank four possible outcomes of a visit to the GP in order of importance. The majority of the participants (44%) ranked Confirmation of UTI Diagnosis as the most important factor, with a mean ranking of 1.71. However, the least prioritized outcome was Obtaining an Antibiotic Prescription (3%), with a mean ranking of 3.04. Table 5 provides a detailed ranking of consultation outcomes, highlighting the number of participants who selected each option, the percentage who deemed it most important, and the corresponding mean scores.

**Table 5:** Ranking of the outcomes of consultation.

Outcome of Consultation	N	% prioritized as the most important	Mean Score
Confirmation of UTI diagnosis	75	44	1.71
Pain relief	48	28	2.49
Obtaining advice on when to contact a GP	43	25	2.74
Obtaining an antibiotic prescription	5	3	3.04

GP = General Practitioner

### 6. Association of level of knowledge regarding UTI with demographics

Distinct trends were found when comparing demographic factors with knowledge levels. Most notably, the age group of 18–30 years showed the highest percentage of high-knowledge individuals (49%) compared to other age groups, while individuals below 18 years predominantly (75%) had poor knowledge about UTIs (p = <0.05). Education level was significantly (p = 0.003) associated with UTI knowledge as Individuals with higher educational qualifications, such as a bachelor’s degree or postgraduate studies, displayed a lower percentage of poor knowledge about UTIs (34% and 21%, respectively). Moreover, the highest percentage (41%) of high-knowledge individuals was found in the postgraduate group. Marital status also exhibited a significant (p = <0.05) association with knowledge, with single individuals demonstrating high levels of knowledge (47%) compared to divorced or married counterparts. While geographical differences in knowledge levels were noted across various Emirates, these were not statistically significant (p = 0.174). The detailed association between participants’ level of knowledge regarding UTIs and their demographic characteristics is clearly presented in Table 6.

**Table 6:** Association of level of knowledge of participants regarding UTI with demographic characteristics.

Variable		Poor Knowledge		Moderate Knowledge		High Knowledge		p-value
	N	n	%	n	%	n	%	
**Age**								**<0.05**
<18	16	12	75	1	6	3	19	
18–30	225	63	28	52	23	110	49	
31–40	113	43	38	44	39	26	23	
41 and above	121	39	32	47	39	35	29	
**Marital Status**								**<0.05**
Divorced	8	4	50	4	50	0	0	
Married	232	78	34	88	38	66	28	
Single	229	73	32	49	21	107	47	
Widow	6	2	33	3	50	1	17	
**Educational Level**								**0.003**
Bachelor’s degree	318	107	34	97	30	114	36	
Post Graduate Studies	66	14	21	25	38	66	28	
High school	86	33	38	20	23	33	38	
Primary/middle school	3	1	33	2	67	0	0	
**Emirate**								**0.174**
Abu Dhabi	88	35	40	25	28	28	32	
Ajman	31	5	16	12	39	14	45	
Dubai	122	45	37	31	25	46	38	
Fujairah	4	1	25	2	50	1	25	
Ras Al Khaimah	3	3	100	0	0	0	0	
Sharjah	225	67	30	74	33	84	37	
Um al Quwain	2	1	50	0	0	1	50	
**Total**	475	157	33	144	30	174	37	

### 7. Association of level of knowledge with attitudes of participants regarding UTI

Individuals with a high level of knowledge about UTIs were significantly (p = <0.05) associated with a greater likelihood of recognizing the seriousness of UTIs (63% of the participants who believed that UTIs are serious and were categorized as having high knowledge also) and their potential complications such as damage to the kidneys and leading to recurrent UTIs.

The belief that UTIs are more common in females was prevalent across all knowledge levels but was most pronounced (52%) in the high-knowledge group. Table 7 provides a detailed overview of how participants’ level of knowledge is associated with their attitudes toward UTIs.

**Table 7:** Association of level of knowledge with attitudes of participants regarding UTI.

Variable		Poor Knowledge		Moderate Knowledge		High Knowledge	
	N	n	%	n	%	n	%
**In your opinion, what is the appropriate way of dealing with UTI?**							
Drink more water	74	24	32	27	36	23	31
Go to the Hospital/Clinic	269	32	12	101	38	136	51
I don’t know	9	2	22	3	33	4	44
Rest at home	1	0	0	0	0	1	100
Shower more frequently	6	1	17	2	33	3	50
Take analgesics (painkillers)	1	0	0	0	0	1	100
Take antibiotics directly	26	9	35	11	42	6	23
**Do you think UTIs are common?**							
Yes	363	64	18	130	36	169	47
No	23	4	17	14	61	5	22
**Which of the following statements do you think is true regarding UTI?**							
I don’t know	34	13	38	16	47	5	15
UTIs affect both genders equally	52	16	31	19	37	17	33
UTIs affect females more	295	39	13	104	35	152	52
UTIs affect males more	5	0	0	5	100	0	0
**Which complications do you expect from having a UTI?**							
It can damage the kidneys	289	37	13	107	37	145	50
It can decrease the quality of life	171	22	13	50	29	99	58
It can lead to recurrent UTIs	142	5	4	33	23	104	73
It can affect pregnancies	85	4	5	18	21	63	74
It can lead to death	29	0	0	2	7	27	93
It can cause weight loss	8	0	0	3	38	5	63
**Do you feel UTI is a serious condition/disease?**							
I don’t know	70	14	20	43	61	13	19
Yes	178	23	13	42	24	113	63
No	138	31	3	59	5	48	4
**Total**	386	68	18	144	37	174	45

### Association of level of knowledge with practices of participants regarding UTI

Higher knowledge levels were associated with more proactive and appropriate health behaviors, such as seeking medical attention promptly and drinking more water. Going to the hospital/clinic was reported 41% with high knowledge but only by 20% of those with poor knowledge. Moreover, a higher proportion of individuals with high knowledge sought medical attention immediately within 24 hours (47%). Drinking more water was a common action across all knowledge levels, but more prevalent in the high-knowledge group (41%). Individuals with higher knowledge tended to drink more water, with 50% of those consuming 1–2 liters having high knowledge and 35% consuming more than 2 liters daily. The relationship between participants’ level of knowledge and their practices regarding UTIs, including the number and percentage of participants, is illustrated in detail in Table 8.

**Table 8:** Association of level of knowledge with practices of participants regarding UTI.

Variable		Poor Knowledge		Moderate Knowledge		High Knowledge	
	N	n	%	n	%	n	%
**Have you ever had a UTI before?**							
Yes	386	68	18	144	37	174	45
No	89	89	100	0	0	0	0
**Total**	475	157	33	144	30	174	37
**What did you do when you felt symptoms of UTI?**							
Drank more water	114	25	22	42	37	47	41
Go to the hospital/clinic	142	29	20	55	39	58	41
Had rest at home	6	0	0	1	17	5	83
Took antibiotics directly	8	0	0	2	25	6	75
Took more showers	5	1	20	3	60	1	20
Took analgesics (painkillers)	19	5	26	7	37	7	37
I don’t remember	2	0	0	1	50	1	50
**Total**	170	37	22	64	38	69	41
**If yes, how long did you wait before visiting the hospital/clinic?**							
< 24 hours	49	8	16	18	37	23	47
24–48 hours	56	14	25	23	41	19	34
2–5 days	32	8	25	12	38	12	38
More than 5 days	18	5	28	8	44	5	28
I did not go to the hospital	17	4	24	3	18	10	59
**Total**	172	39	23	64	37	69	40
**Which symptoms do you have?**							
Pain during urination	129	24	19	48	37	57	44
Change in the urine color and appearance	83	13	16	33	40	37	45
Back pain	55	10	18	22	40	23	42
Increase in the frequency of urination	94	12	13	36	38	46	49
A sudden desire to go to bathroom to urinate	87	16	18	31	36	40	46
Fever	33	3	9	15	45	15	45
Abdominal pain	67	15	22	23	34	29	43
**Total**	167	36	22	62	37	69	41
**How much water do you drink per day?**							
< 0.5 Liters	34	8	24	16	47	10	29
0.5 Liters to 1 Liter	159	24	15	60	38	75	47
1 Liter to 2 Liters	142	24	17	47	33	71	50
More than 2 Liters	51	12	24	21	41	18	35
**Total**	386	68	18	144	37	174	45

## Discussion

Urinary tract infections (UTI) are one of the most common, challenging, and misunderstood diseases that women experience as outpatients [13]. UTIs are a prevalent health issue among females globally [1], with a notable prevalence in the United Arab Emirates (UAE), which is increasing at an exponential rate [14]. The issue of delayed detection of UTIs is very prevalent. It leads to both excessive therapy and inadequate treatment [13]. If not managed properly and timely or left untreated, UTIs may cause serious complications like renal impairment, kidney scarring, sepsis, and pyelonephritis [5]. Women’s practices, attitudes, and understanding of urinary tract infections are significant variables that can affect how this illness is managed and prevented. While a substantial number of studies on this subject have been carried out globally [9, 10, 13], there remains no data specific to the UAE context. This study aims to bridge this gap by exploring the knowledge, attitudes, and practices of women in the United Arab Emirates about urinary tract infections (UTIs).

Our research revealed several noteworthy findings. Most of the participants (80%) were aware of the term UTI and half of them were able to identify the correct definition. The vast majority were able to identify the most common cause of UTIs and the most prevalent symptoms. Additionally, most of them were also able to report the most appropriate ways of dealing with UTI. Therefore, the results of our study concluded that most of the respondents exhibited an acceptable level of awareness regarding UTIs. Our results regarding the knowledge of the participants are consistent with the other studies in the surrounding region like in Saudi Arabia [10, 15], and were also consistent with a study conducted in the Netherlands [9]. On the other hand, studies conducted in Bangalore [16] and Egypt [17] suggested that the participants mostly had poor knowledge and attitude, which may suggest that lower levels of knowledge are associated with developing countries. Similarly, while assessing the attitudes and practices of the respondents, we found out that a significant fraction of our study population understood the seriousness of the condition (46%) and took appropriate measures for the management of UTIs, such as seeking medical help (48%) or increasing water intake (38.5%). When asked about the outcomes of General Practitioner (GP) consultation, the participants in our study showed results similar to those of the study group in the Netherlands from Cox SML et al. [9]. In both studies, most participants chose confirmation of UTI diagnosis as the main outcome behind the GP visit, which shows that patients value getting the definitive diagnostic information during their consultation. Moreover, in our study, only 3% of the participants prioritized obtaining an antibiotic prescription compared to 14.3% in the Netherlands study [9].

These findings may be explained by the wider accessibility of antibiotics without a prescription in the UAE. Uropathogenic bacteria are notable for their frequent resistance to many drugs [18]. It is necessary to monitor the antibiotic resistance patterns in UTIs [19], as evidenced by a recent study conducted in Sharjah, which revealed that trimethoprim-sulfamethoxazole, cephalexin, and amoxicillin—commonly used as first-line antimicrobials—are becoming increasingly ineffective in treatment of UTIs. Therefore, alternative treatment regimens should be considered, and further monitoring of antibiotic resistance patterns in the UAE is essential. the use of fluoroquinolones was found as a preferable choice for empirical therapy [20].

Regarding patients’ preferences for antibiotic usage during UTIs, participants were asked about the appropriate way to manage a urinary tract infection. Only 6.7% of the participants chose to take antibiotics immediately, while just 2.7% took antibiotics after experiencing the initial UTI symptoms. This indicates that only a total of 9.4% of the participants were inclined to take antibiotics before receiving a diagnosis. These results suggest increased awareness of appropriate antibiotic use and a preference for more conservative treatment approaches among the UAE population. Moreover, this trend aligns with global health concerns regarding antibiotic resistance, signifying a positive step towards responsible antibiotic utilization within the UAE community.

Furthermore, we studied the association of knowledge level and demographics, attitudes, and practices and concluded that the individuals who possessed a high level of knowledge were 18–30 years old and were well-educated. A similar study was conducted in Malaysia, which stated a possibility that educated respondents most likely would have studied about UTI or UTI-related topics in their syllabus, which could be a possible reason for their high level of knowledge [21]. These individuals also understood the seriousness of UTIs and preferred to seek medical help for treatment. Significant associations were found between knowledge and age, educational level, and marital status; however, no significant association was found between the level of knowledge and Emirate which is in contrast to studies conducted in Saudi Arabia [10, 15].

However, certain strengths and limitations of our study need to be acknowledged. This is the first study that assessed the knowledge, attitudes, and practices of women regarding UTIs in the UAE. Enhancing patients’ understanding, beliefs, and actions about prevalent illnesses plays a crucial part in health education [22]. The results of this study can guide the tailored educational campaigns for women’s health in the UAE. The study’s limitations include the possible occurrence of recall bias due to the dependence on self-reported data, as well as the possibility of participants’ replies being influenced by social desirability or response bias.

In conclusion, our study found that most of the respondents had adequate knowledge about UTIs and they believed that going to a hospital/clinic is the appropriate way to deal with a UTI, which suggests that most of the women in the UAE exhibited substantial awareness and behavioral patterns regarding UTIs. Women of the age 18–30 years comprised the highest percentage of high-knowledge level individuals. Higher knowledge levels were associated with higher educational qualification and more proactive and appropriate health behaviors, such as seeking medical attention promptly & drinking more water. Continuous medical education can significantly contribute to enhancing understanding and attitudes towards urinary tract infections (UTIs) in order to minimize avoidable outcomes.
